# Concordance between clinician-reported toxicities and patient-reported symptom severity in early-stage breast cancer: an analysis of the randomized phase III PANTHER trial

**DOI:** 10.1016/j.esmoop.2026.107696

**Published:** 2026-05-09

**Authors:** P. Filis, X. Liu, A. Matikas, M. Untch, G.G. Steger, H. Johansson, M. Hellström, M. Gnant, S. Loibl, Y. Brandberg, J. Bergh, T. Foukakis

**Affiliations:** 1 Department of Oncology/Pathology, Karolinska Institutet, Stockholm, Sweden; 2 Breast Center, Karolinska Comprehensive Cancer Center and Karolinska University Hospital, Stockholm, Sweden; 3 MSB Medical School Berlin, Helios Klinikum Berlin-Buch, Berlin, Germany; 4 Department of Internal Medicine I, Medical University of Vienna, Vienna, Austria; 5 Austrian Breast and Colorectal Cancer Study Group, Vienna, Austria; 6 Comprehensive Cancer Center, Medical University of Vienna, Vienna, Austria; 7 German Breast Group, Neu-Isenburg, Germany

**Keywords:** breast cancer, CTCAE, health-related quality of life, patient-reported outcomes, toxicity

## Abstract

**Background:**

Although advances in systemic therapy have markedly improved survival in breast cancer, treatment-related toxicities remain a major determinant of health-related quality of life (HRQoL). Clinician reports may not fully capture the symptom burden experienced by patients. This study examined the concordance between clinician-reported toxicities and patient-reported symptom scores during adjuvant chemotherapy for early-stage, high-risk breast cancer in a randomized phase III clinical trial.

**Patients and methods:**

Clinician-reported toxicities were graded using the Common Terminology Criteria for Adverse Events (CTCAE) version 3.0, and patient-reported symptom scores were obtained with the European Organisation for Research and Treatment of Cancer Quality of Life Questionnaire Core 30 (EORTC QLQ-C30) and EORTC QLQ-BR23 questionnaires at mid- and end-of-treatment. Analyses focused on six symptomatic domains with conceptual overlap between CTCAE and EORTC measures: diarrhea, nausea, vomiting, mucositis, pain, and fatigue. Concordance between clinician- and patient-reported scores was evaluated using weighted Cohen’s kappa coefficients.

**Results:**

A total of 1566 patients with available CTCAE and HRQoL data were included in the analysis. Agreement between clinician-reported toxicities and patient-reported symptom scores was low across all symptoms and time points, with kappa values indicating no to minimal concordance (kappa = 0.07-0.34). Clinician-assigned CTCAE grades were predominantly lower than patient-reported symptom severity. The highest discrepancy rates were observed for fatigue (36%-54%) and pain (23%-38%) across treatment arms and time points. Except for nausea and vomiting, the magnitude of the discrepancy increased from mid- to end-of-treatment in both study arms. Binary analyses comparing the presence of any versus no symptom confirmed persistently low agreement between clinicians and patients (kappa = 0.03-0.35).

**Conclusion:**

Clinician-reported toxicities showed poor concordance with patient-reported symptom severity, and the discrepancy increased over time. Incorporating patient-reported assessments into both clinical trials and routine care is essential to ensure more accurate evaluation of treatment tolerability.

## Highlights

• Poor concordance was observed between clinician-reported toxicities and patient-reported symptoms (kappa = 0.07-0.34).
• Symptom severity was systematically rated lower by clinicians than by patients.
• Fatigue and pain showed the largest clinician–patient reporting gaps.
• The discrepancy increased from mid- to end-of-treatment for most outcomes and for both treatment arms.

## Introduction

Breast cancer remains the most commonly diagnosed malignancy among women, with incidence steadily increasing over the past decade.^1^ Nevertheless, the evolution of systemic therapies, coupled with the refinement of screening, surgical, and radiation procedures, has led to durable oncological responses in this cancer type.^2^ As the population of breast cancer survivors continues to expand, attention has increasingly shifted from survival alone toward maintaining and improving health-related quality of life (HRQoL) during and after treatment.^3^ Chemotherapy remains a cornerstone of systemic therapy in early-stage disease, with sequential anthracycline- and taxane-based regimens widely adopted for high-risk populations.^2^^,^^4^ However, these regimens are associated with a broad range of acute and chronic toxicities that may compromise treatment adherence, long-term outcomes, and patients’ HRQoL.^5^

Systematic evaluation of treatment-related toxicities is essential to ensure both efficacy and tolerability of therapy. The Common Terminology Criteria for Adverse Events (CTCAE), developed by the National Cancer Institute, is used in both clinical trials and routine practice to classify and grade the severity of treatment-related adverse effects (AEs).^6^ In parallel, there has been growing emphasis on integrating patient-reported outcomes (PROs), which provide unique insight into the lived experience of cancer treatment and its impact on daily functioning.^7^ PROs can be captured using instruments that focus specifically on symptomatic toxicities, such as the Patient-Reported Outcomes version of the CTCAE (PRO-CTCAE),^8^ or broader HRQoL questionnaires, such as the European Organisation for Research and Treatment of Cancer Quality of Life Questionnaire Core 30 (EORTC QLQ-C30) and its disease-specific modules.^9^ Although PRO-CTCAE mirrors clinician terminology from the patient perspective, the EORTC questionnaires assess functional domains, symptom burden, and overall HRQoL.^10^ Importantly, growing evidence indicates that clinician-reported CTCAE grades may underrepresent the symptom burden experienced by patients, highlighting the need to better understand the concordance between clinician assessments and patient-reported HRQoL.^11^^,^^12^

The PANTHER trial was an open-label, randomized, multicenter phase III study that evaluated whether tailored dose-dense adjuvant chemotherapy improves outcomes in nonmetastatic breast cancer compared with a conventional three-weekly chemotherapy schedule.^13^^,^^14^ Leveraging this dataset, the primary objective of this study was to evaluate the concordance between CTCAE and patient-reported symptom scores (EORTC instruments) in patients with early-stage, high-risk breast cancer during treatment. In addition, we aimed to characterize the discrepancies between clinician- and patient-reported scores and to explore how this discordance may change over the course of treatment.

## Patients and methods

### Study design

The PANTHER trial (NCT00798070) was a prospective, multicenter, open-label, phase III randomized study conducted across 86 sites in Sweden, Germany, and Austria. Ethical approval was obtained from institutional review boards at all participating centers, and written informed consent was secured from all patients before enrollment. The study was conducted in accordance with the Declaration of Helsinki and principles of Good Clinical Practice.

### Patient population and treatment regimens

The design and eligibility criteria of the PANTHER trial have been described in detail elsewhere.^13^ In summary, participants were women, aged 65 years or younger, with early-stage breast cancer, who had undergone surgical resection for node-positive disease or high-risk node-negative disease (defined as tumor grade 3, diameter larger than 2 cm, and estrogen receptor (ER)-negative status, or age <35 years). All patients had an Eastern Cooperative Oncology Group performance status of 0 or 1. Participants were randomly assigned in a 1:1 ratio to either four cycles of tailored and dose-dense adjuvant epirubicin and cyclophosphamide every 2 weeks, followed by four cycles of tailored dose-dense docetaxel every 2 weeks (experimental arm) or to standard-interval chemotherapy with three cycles of fluorouracil and epirubicin-cyclophosphamide every 3 weeks, followed by three cycles of docetaxel every 3 weeks (standard arm). Dose tailoring for each cycle in the experimental group was decided according to a predefined algorithm based on hematologic and nonhematologic toxicities.^13^ Patients with tumors overexpressing human epidermal growth factor receptor 2 (HER2) were treated with adjuvant trastuzumab for a duration of 1 year. Those with ER-positive breast cancer received endocrine therapy, either tamoxifen or an aromatase inhibitor with or without ovarian function suppression, for a minimum of 5 years, initiated following completion of chemotherapy. Posttreatment follow-up included routine clinical evaluations as well as hematologic and biochemical monitoring.

### Patient-reported HRQoL

Patients were informed, orally and in writing, about the HRQoL study before inclusion into the clinical study. HRQoL assessments were conducted at baseline (before randomization), midtreatment (cycle 4 for experimental arm and cycle 3 for standard arm), end-of-treatment (cycle 8 for experimental arm and cycle 6 for standard arm), and at 4-, 8-, and 12-month follow-up. Patient-reported HRQoL was assessed using the European Organisation for Research and Treatment of Cancer Quality of Life Questionnaire Core 30 (EORTC QLQ-C30, version 3.0), a validated instrument designed to evaluate multiple dimensions of HRQoL in cancer patients.^9^ EORTC QLQ-C30 comprises 30 items covering functional scales as well as symptom scales and single items assessing symptom burden. Most items are scored on a four-point Likert scale (1 as ‘not at all,’ 2 as ‘a little,’ 3 as ‘quite a bit,’ 4 as ‘very much’), whereas global health status/overall HRQoL is rated on a seven-point scale. In addition, the EORTC Breast Cancer Module (EORTC QLQ-BR23) was administered in conjunction with the EORTC QLQ-C30 to capture breast cancer-specific concerns.^15^ The EORTC BR-23 consists of 23 items grouped into four functional scales and four symptom scales. All items are rated in the same four-point response format as the core questionnaire. The items refer to symptoms experienced during the preceding week, thereby capturing the recent symptom burden.

### Clinician-reported adverse events

Toxicities were assessed by clinicians before each cycle for any adverse events that were experienced during the previous cycle. Clinician-reported adverse events were evaluated using the CTCAE, a standardized tool developed by the National Cancer Institute to classify and grade the severity of treatment-related toxicities in clinical trials.^6^ A grade of 0 is defined as ‘no AE,’ 1 as ‘mild AE,’ 2 as ‘moderate,’ 3 as ‘severe,’ 4 as ‘life-threatening,’ and 5 as ‘death related to AE.’ CTCAE version 3.0 was used in this study, as the trial enrolled patients from February 2007 to September 2011, in which grade 1 indicates a mild AE, grade 2 a moderate AE, grade 3 a severe AE, grade 4 a life-threatening AE, and grade 5 death related to the AE. The toxicities evaluated included diarrhea, nausea, vomiting, mucositis, febrile neutropenia, infection, pain, fatigue, neuropathy, and nail changes.

To evaluate the alignment of clinician-reported CTCAE and patient-reported HRQoL, we a priori selected symptomatic adverse events with direct conceptual overlap in the EORTC instruments, including diarrhea (‘have you had diarrhea’), nausea (‘have you felt nauseated’), vomiting (‘have you vomited’), mucositis (‘did you have a dry mouth’), pain (‘have you had pain,’ ‘did pain interfere with your daily activities’), and fatigue (‘did you need to rest,’ ‘have you felt weak,’ ‘were you tired’) (Supplementary Table S1, available at https://doi.org/10.1016/j.esmoop.2026.107696). This approach ensured comparability between clinician- and patient-reported measures by focusing on symptoms captured using analogous constructs across instruments.

In the PANTHER trial, matched CTCAE and HRQoL data were available at two time points: midtreatment and end-of-treatment. CTCAE grading was carried out on a scale from 0 to 4, whereas EORTC items were coded on a one- to four-point scale. For multi-item domains (fatigue and pain), raw scores were linearly transformed to 0-100 scales according to the scoring manual^16^ and then categorized into four severity levels to align with the single-item scales. For these multi-item domains, scoring followed the EORTC guidelines, which require that at least half of the items in each scale have to be completed in order to impute missing values; otherwise, the scale score was considered as missing.

### Statistical analysis

For each symptom, the matched cases at the middle and end of treatment were identified and summarized by frequency. The assessments for the middle and end of treatment correspond to cycles 4 and 8 for the tailored dose-dense treatment arm, and cycles 3 and 6 for the standard-interval chemotherapy arm, respectively. Concordance between clinician- and patient-reported outcomes was classified as agreement, underreporting (clinician grade lower than patient score), or overestimation (clinician grade higher than patient score). Specifically, agreement was defined as any of the following four pairs of scales, according to previously published literature: CTCAE grade 0 and EORTC score 1, CTCAE grade 1 and EORTC score 2, CTCAE grade 2 and EORTC score 3, CTCAE grades 3 and 4 combined, and EORTC score 4 (Supplementary Table S2, available at https://doi.org/10.1016/j.esmoop.2026.107696).^17^ The extent of four-level agreement was quantified using weighted Cohen’s kappa coefficients (quadratic weights) with corresponding *P* values. In addition to the ordinal four-level analyses, a binary analysis was also carried out, recoding CTCAE as none (grade 0) versus any grade (grade ≥1) and EORTC as none (score 1) versus any severity (scores 2-4).^18^ For each symptom, 2 × 2 contingency tables were constructed to classify cases as ‘reported by neither,’ ‘reported only by clinician,’ ‘reported only by patient,’ or ‘reported by both.’ Concordance in the binary analysis was assessed using unweighted Cohen’s kappa. Interpretation followed conventional thresholds: 0-0.20 no agreement, 0.21-0.39 minimal, 0.40-0.59 weak, 0.60-0.79 moderate, 0.80-0.90 strong, and >0.90 almost perfect agreement.^19^ All these analyses were stratified by treatment arm [tailored dose-dense (arm A) and standard-interval (arm B) chemotherapy] and time point (mid- and end-of-treatment).

Further, for discordant cases, the relative frequency of underreporting versus overestimation was described, and an exact binomial test was used to test for predominance of lower clinician-assigned grades compared to patient-reported symptom severity. Statistical significance was defined as two-sided *P* < 0.004, taking multiple testing into account. Changes in discordance from mid- to end-of-treatment within each arm were tested using McNemar’s test, restricted to patients with paired data at both time points. Between-arm comparisons at a given time point were carried out using two-sample tests for proportions. All analyses were conducted in R, version 4.5.1 (R Foundation for Statistical Computing, Vienna, Austria).

## Results

A total of 1566 patients with early-stage, high-risk breast cancer and available CTCAE and HRQoL data were included in the present analysis (Table 1). Matched clinician-reported toxicities and patient-reported EORTC symptom scores were assessed at two time points: midtreatment and end-of-treatment. The midtreatment assessment corresponded to the end of the fourth chemotherapy cycle in arm A (*N* = 674) and the end of the third cycle in arm B (*N* = 711). The end-of-treatment assessment corresponded to the end of the eighth cycle for arm A (*N* = 594) and the end of the sixth cycle for arm B (*N* = 699). Analyses were separately carried out on six symptom scales with direct conceptual overlap between CTCAE and EORTC measures: diarrhea, nausea, vomiting, mucositis, pain, and fatigue.

**Table 1:** Characteristics for study participants with available data for both clinician-reported toxicities and patient-reported symptom severity, stratified by original clinical trial arms

	Tailored dose-dense chemotherapy (arm A)	Standard chemotherapy (arm B)
No. of patients	755	811
Age, years, median (Q1-Q3)	51.1 (45.1-57.7)	50.2 (44.4-57.7)
No. of positive nodes		
0	24 (3.2%)	22 (2.7%)
1-3	448 (59.3%)	445 (54.9%)
4-9	199 (26.4%)	237 (29.2%)
>9	84 (11.1%)	107 (13.2%)
Tumor size, cm		
≤2	308 (41.1%)	329 (40.6%)
2-5	385 (51.3%)	427 (52.7%)
>5	57 (7.6%)	54 (6.7%)
Missing	5	1
Hormone receptor status		
ER− & PR−	148 (19.6%)	166 (20.5%)
ER+/PR+	607 (80.4%)	645 (79.5%)
HER2 status		
Negative	626 (82.9%)	670 (82.6%)
Positive	129 (17.1%)	141 (17.4%)
Tumor grade		
I	46 (6.1%)	45 (5.5%)
II	361 (47.9%)	412 (50.8%)
III	346 (45.9%)	354 (43.6%)
Missing	2	0
Molecular subtype		
HER2-positive	129 (17.1%)	141 (17.4%)
HR+/HER2-negative	524 (69.4%)	564 (69.5%)
TNBC	102 (13.5%)	106 (13.1%)
Middle of treatment (no.)		
Available CTCAE data	955	983
Available CTCAE and QoL data	674	711
End of treatment (no.)		
Available CTCAE data	801	913
Available CTCAE and QoL data	594	699

CTCAE, Common Terminology Criteria for Adverse Events; ER, estrogen receptor; HER2, human epidermal growth factor receptor 2 gene; TNBC, triple-negative breast cancer; QoL, quality of life; PR, progesterone receptor.

Agreement between clinician-reported CTCAE toxicities and patient-reported EORTC symptom scores was none to limited across all symptoms and time points (Table 2).^19^ At midtreatment, weighted Cohen’s kappa values in arm A ranged from 0.14 for mucositis to 0.37 for diarrhea, and in arm B from 0.07 for pain to 0.26 for nausea and vomiting. At end-of-treatment, kappa values ranged from 0.07 for mucositis to 0.31 for vomiting in arm A, and from 0.04 for mucositis to 0.34 for vomiting in arm B.

**Table 2:** Agreement between clinician-reported CTCAE toxicities and patient-reported EORTC symptom scores, with weighted Cohen’s kappa presented by treatment arm and time point

CTCAE toxicities/EORTC items	No.	Cohen’s kappa^a^	*P* value	No.	Cohen’s kappa^a^	*P* value	No.	Cohen’s kappa^a^	*P* value	No.	Cohen’s kappa^a^	*P* value
	Middle of treatment						End of treatment					
	Arm A			Arm B			Arm A			Arm B		
Diarrhea	666	0.37	<0.001	705	0.23	<0.001	587	0.27	<0.001	696	0.24	<0.001
Nausea	667	0.35	<0.001	705	0.26	<0.001	590	0.30	<0.001	698	0.30	<0.001
Vomiting	671	0.30	<0.001	709	0.26	<0.001	589	0.31	<0.001	699	0.34	<0.001
Mucositis (oral)	664	0.14	<0.001	704	0.09	<0.001	586	0.07	<0.001	688	0.04	<0.001
Fatigue	653	0.19	<0.001	698	0.14	<0.001	583	0.20	<0.001	689	0.21	<0.001
Pain	658	0.20	<0.001	697	0.07	<0.001	581	0.25	<0.001	687	0.18	<0.001

CTCAE, Common Terminology Criteria for Adverse Events; EORTC, European Organisation for Research and Treatment of Cancer.

a Cohen’s kappa interpretation follows conventional thresholds (McHugh, 2012^19^): 0-0.20 no agreement, 0.21-0.39 minimal, 0.40-0.59 weak, 0.60-0.79 moderate, 0.80-0.90 strong, and >0.90 almost perfect agreement.

Based on the predefined interpretation thresholds, mucositis, fatigue, and pain demonstrated, no agreement between clinician and patient assessments, whereas diarrhea, nausea, and vomiting showed minimal agreement at both time points. The magnitude of agreement was comparable between treatment arms across time points. Only for fatigue, the agreement was significantly lower in arm A than in arm B for both time points (*P* < 0.001 and 0.021, respectively).

Overall, the low kappa values observed across all symptoms reflected substantial clinician-patient discordance. This discordance is primarily driven by clinician-assigned grades lower than patient-reported scores (Supplementary Table S3, available at https://doi.org/10.1016/j.esmoop.2026.107696). Across symptoms and time points, binomial tests demonstrated a statistically significant predominance of lower clinician-assigned grades (*P* < 0.001), except for vomiting in arm B at midtreatment (Supplementary Table S3, available at https://doi.org/10.1016/j.esmoop.2026.107696). Overestimation was rare, occurring in fewer than 5% of observations across all symptoms.

**Figure 1:**
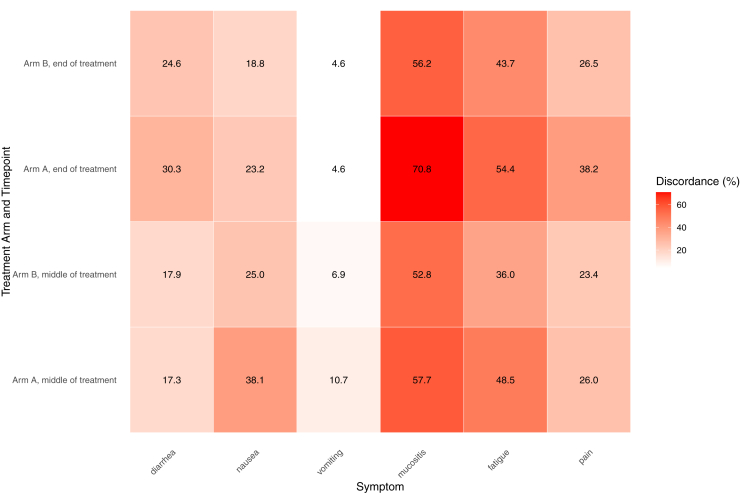
**Discordance between clinician-assigned toxicity grading and patient-reported symptoms.** Heatmap of the proportion of cases in which clinician-assigned CTCAE grades were lower than patient-reported EORTC symptom scores, stratified by treatment arm and timepoint (middle versus end of treatment). Each cell shows the percentage of underreporting for the corresponding symptom, with lighter shades of red indicating lower rates and darker shades indicating higher rates (observed range: 4.6%-70.8%). The figure highlights that discordance was most pronounced for mucositis, fatigue, and pain. CTCAE, Common Terminology Criteria for Adverse Events; EORTC, European Organisation for Research and Treatment of Cancer.

At midtreatment, the proportion of patients for whom clinicians reported lower grades ranged from 10.7% for vomiting to 57.7% for mucositis in arm A, and from 6.9% for vomiting to 52.8% for mucositis in arm B (Figure 1, Supplementary Table S3, available at https://doi.org/10.1016/j.esmoop.2026.107696). At the end of treatment, underreporting ranged from 4.6% for vomiting to 70.8% for mucositis in arm A, and from 4.6% for vomiting to 56.2% for mucositis. Fewer differences were seen for vomiting and diarrhea, whereas mucositis, fatigue, pain, and nausea showed higher discrepancy (Figure 1). Discordance was significantly more frequent in arm A for nausea (*P* < 0.001), vomiting (*P* = 0.016), and fatigue (*P* < 0.001) at midtreatment, and for diarrhea (*P* = 0.025), mucositis (*P* < 0.001), fatigue (*P* < 0.001), and pain (*P* < 0.001) by the end of treatment.

**Figure 2:**
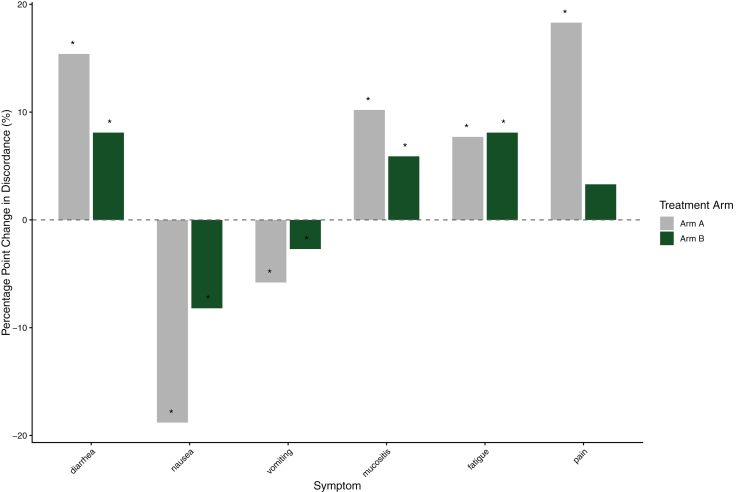
**Change in discordance between clinician-assigned toxicity and patient-reported symptom severity from mid- to end-of-treatment.** Bars show the percentage point change in discordance rates (clinician-reported CTCAE grade lower than the corresponding EORTC symptom score) between mid- and end-of-treatment, stratified by treatment arm. Positive values indicate an increase in underreporting from the middle to end of treatment, whereas negative values indicate a decrease. Asterisks denote statistical significance from McNemar’s test (*P* < 0.05), based on paired patients with available data at both time points. CTCAE, Common Terminology Criteria for Adverse Events; EORTC, European Organisation for Research and Treatment of Cancer.

Among patients with clinician- and patient-reported data available at both mid- and end-of-treatment, discordance changed over time (Figure 2). From mid- to end-of-treatment, clinician underreporting decreased only for nausea and vomiting in both treatment arms. In contrast, this discrepancy increased for all other symptoms, with statistically significant within-arm changes observed across both arms. The largest increases were noted for diarrhea and pain in arm A (15% and 18% increase, respectively).

In a binary concordance analysis comparing the presence of none versus any symptom, again, agreement between clinician- and patient-reported outcomes remained low across all symptom domains (Table 3). Patient-only reporting was consistently more common than clinician-only reporting. Across all symptoms, Cohen’s kappa coefficients indicated none to minimal agreement (kappa range: 0.03-0.35), in line with the results from the ordinal-scale analysis. Concordance between clinician-assigned CTCAE grades and patient-reported symptom severity stratified by participating country is presented in Supplementary Table 4 (available at https://doi.org/10.1016/j.esmoop.2026.107696).

**Table 3:** Concordance between clinician-reported toxicities (any grade versus none) and patient-reported symptoms (any severity versus none)

Treatment arm and time point	Toxicity	Number of patients	Reported by neither patient nor physician No. (%)	Reported by physician but not patient No. (%)	Reported by patient but not physician No. (%)	Reported by both patient and physician No. (%)	Cohen’s kappa
Arm A, middle of treatment	Diarrhea	666	495 (74.3)	27 (4.1)	95 (14.3)	49 (7.4)	0.35
	Nausea	667	149 (22.3)	76 (11.4)	155 (23.2)	287 (43)	0.29
	Vomiting	671	522 (77.8)	55 (8.2)	64 (9.5)	30 (4.5)	0.23
	Mucositis	664	95 (14.3)	37 (5.6)	265 (39.9)	267 (40.2)	0.13
	Fatigue	653	38 (5.8)	73 (11.2)	129 (19.8)	413 (63.2)	0.09
	Pain	658	310 (47.1)	95 (14.4)	145 (22)	108 (16.4)	0.20
Arm B, middle of treatment	Diarrhea	705	504 (71.5)	46 (6.5)	114 (16.2)	41 (5.8)	0.21
	Nausea	705	206 (29.2)	182 (25.8)	98 (13.9)	219 (31.1)	0.22
	Vomiting	709	573 (80.8)	69 (9.7)	41 (5.8)	26 (3.7)	0.24
	Mucositis	704	150 (21.3)	54 (7.7)	303 (43)	197 (28)	0.09
	Fatigue	698	104 (14.9)	116 (16.6)	165 (23.6)	313 (44.8)	0.12
	Pain	697	365 (52.4)	108 (15.5)	153 (22)	71 (10.2)	0.09
Arm A, end of treatment	Diarrhea	587	326 (55.5)	26 (4.4)	147 (25)	88 (15)	0.33
	Nausea	590	378 (64.1)	37 (6.3)	121 (20.5)	54 (9.2)	0.25
	Vomiting	589	551 (93.5)	8 (1.4)	23 (3.9)	7 (1.2)	0.29
	Mucositis	586	49 (8.4)	23 (3.9)	289 (49.3)	225 (38.4)	0.05
	Fatigue	583	26 (4.5)	42 (7.2)	130 (22.3)	385 (66)	0.08
	Pain	581	131 (22.5)	73 (12.6)	152 (26.2)	225 (38.7)	0.22
Arm B, end of treatment	Diarrhea	696	421 (60.5)	48 (6.9)	149 (21.4)	78 (11.1)	0.27
	Nausea	698	487 (69.8)	39 (5.6)	115 (16.5)	57 (8.2)	0.30
	Vomiting	699	649 (92.8)	12 (1.7)	30 (4.3)	8 (1.1)	0.25
	Mucositis	688	106 (15.4)	64 (9.3)	300 (43.6)	218 (31.7)	0.03
	Fatigue	689	61 (8.9)	69 (10)	151 (21.9)	408 (59.2)	0.16
	Pain	687	165 (24)	178 (25.9)	135 (19.7)	209 (30.4)	0.09

## Discussion

This study examined the concordance between clinician-reported toxicities using CTCAE and patient-reported symptom severity using EORTC instruments in patients with nonmetastatic, high-risk breast cancer enrolled in a randomized phase III trial. Across all symptom domains, agreement between clinicians and patients was low, with Cohen’s kappa values indicating no to minimal concordance. This discrepancy is primarily driven by clinician underreporting, reflecting the inherent limitations of clinician-based grading systems in capturing the full extent of patients’ symptom burden. These findings underscore the importance of systematically integrating PROs into toxicity monitoring to complement clinician evaluations and achieve a more comprehensive assessment of the patient experience during cancer treatment.

Across individual symptoms, notable differences emerged in the degree of concordance. Subjective symptoms such as fatigue and pain exhibited the lowest agreement between clinicians and patients, whereas more objectively quantifiable symptoms like vomiting and diarrhea demonstrated comparatively higher concordance. These findings are consistent with prior research indicating that clinician assessments tend to align more closely with patient reports for observable or measurable toxicities, whereas subjective and multidimensional symptoms are more frequently underrecognized by healthcare providers.^20^^,^^21^ Thus, discrepancies may be driven by clinicians’ reliance on observable or clinically prioritized toxicities, as well as by differences in symptom perception between patients and clinicians, particularly for multidimensional symptoms such as fatigue and pain. Furthermore, discordance increased over time for most symptoms, indicating a growing divergence between clinician and patient perspectives as treatment progressed. This pattern likely reflects the cumulative burden of therapy and the inherent limitations of clinician-based assessment tools, such as CTCAE, in capturing the evolving patient experience. One possible explanation is that patients may have more frequent contact with clinicians during treatment, allowing more opportunities for symptom discussion compared with the end of treatment, when such interactions may be less frequent. Importantly, in our study, ∼18% of patients in the experimental arm prematurely discontinued treatment, of whom half did not receive the final cycle of therapy, highlighting how underappreciated toxicity may ultimately affect treatment delivery.^13^ The observed trend underscores the need for sustained vigilance in toxicity monitoring throughout treatment and reinforces the value of systematically incorporating patient-reported outcome measures into clinical trials, where they can provide a more sensitive and continuous assessment of cumulative treatment-related burden and its impact on HRQoL.

Prior investigations have also raised concerns regarding discordance between clinician- and patient-reported outcomes in oncology. Two studies comparing toxicities and EORTC QLQ-C30 data in clinical trials across various cancer types, including breast cancer, demonstrated a high risk of underreporting by physicians.^17^^,^^18^ Notably, one study identified substantial discrepancies in physician reporting specifically for fatigue.^17^ Similarly, studies evaluating the agreement between clinician-reported CTCAE and patient-reported PRO-CTCAE outcomes in breast cancer populations have revealed low concordance.^10^^,^^22^ In one analysis, nurse-reported adverse events were found to align more closely with patient reports than physician-reported events.^22^ Moreover, symptoms such as fatigue, anxiety, and discouragement were more readily captured by PRO-CTCAE than by traditional CTCAE assessments.^10^ In contrast, consistency between EORTC QLQ-C30 and PRO-CTCAE has been demonstrated in heterogeneous cancer populations, supporting the complementary value of these patient-reported instruments.^23^

The discrepancies observed between clinician- and patient-reported outcomes in our study and in the published literature underscore the complementary nature of these assessment approaches in evaluating treatment-related toxicity. Although CTCAE grading offers a standardized clinical framework, PROs capture subjective symptom burden that may not be readily apparent to healthcare providers. Consequently, the modest correlations between these measures likely reflect their focus on different dimensions of the symptom experience.^24^ Accordingly, the systematic integration of patient-reported outcome measures, such as PRO-CTCAE or HRQoL instruments, into contemporary clinical trials is increasingly recognized as essential for comprehensive toxicity evaluation.^25^ Furthermore, a recently published multinational randomized controlled trial showed that providing clinicians with real-time patient-reported outcome data during CTCAE grading significantly improved interrater reliability for most symptomatic adverse events.^26^ As breast cancer treatment regimens grow increasingly complex, the integration of at least one form of patient-centered assessment into both clinical research and routine care is essential to fully capture treatment tolerability and patient well-being.

Existing evidence on the concordance between CTCAE and PROs in breast cancer is limited, often derived from pooled analyses of randomized trials in which breast cancer represents only a small subset of cases^17^^,^^18^ or from nontrial settings.^10^^,^^22^ A key strength of this study is the use of prospectively collected PROs alongside clinician-reported toxicity grading within the context of a large, multicenter, randomized phase III trial. The opportunity to compare a conventional chemotherapy schedule with a more intensified dose-dense approach in a younger breast cancer population enhances the robustness and clinical relevance of our findings. Furthermore, the use of both ordinal four-level and binary concordance analyses enhances the methodological rigor of the study, whereas assessing the pattern of discordance over time provides an insightful perspective for trial-based assessments that is currently lacking in this field. However, certain limitations should also be acknowledged. First, toxicity was assessed using CTCAE version 3.0, which was available at the time of trial initiation, although newer versions provide more nuanced toxicity definitions. Nevertheless, studies using more recent CTCAE versions have reported similar levels of concordance between clinician- and patient-reported outcomes, suggesting that our findings remain broadly comparable.^22^ Second, concordance was assessed at only two time points, limiting our ability to explore temporal trends in greater detail or to evaluate changes beyond the treatment period. Furthermore, it should be noted that the mapping of mucositis to the EORTC item ‘dry mouth’ represents an approximation, as these are not identical clinical entities, but capture related symptomatic aspects. In addition, grouping CTCAE grades 3-4 with EORTC score 4 is justified by the low frequency of high-grade events and supported by prior literature,^17^ although this approach may have modestly inflated agreement estimates. Finally, the generalizability of these results to older patients, who may experience different toxicity profiles and HRQoL trajectories, should be approached with caution.

In conclusion, there are substantial discrepancies between clinician- and patient-reported toxicities during adjuvant chemotherapy for early-stage breast cancer, with clinicians consistently underreporting the severity of patient-reported symptoms. Incorporating systematic patient-reported assessments into both clinical trials and routine clinical practice is essential to improve symptom recognition, enhance patient-clinician communication, and ultimately optimize the quality of supportive care in breast cancer management.
