# Supplementary material for: Concordance between clinician-reported toxicities and patient-reported symptom severity in early-stage breast cancer: an analysis of the randomized phase III PANTHER trial
Source: ESMO Open. 2026 May 9;11(5):107696. doi: 10.1016/j.esmoop.2026.107696 (PMC13242076; doi:10.1016/j.esmoop.2026.107696)
Supplement: Supplementary Table S1-S4 [file mmc1.docx]

**Supplementary Material**

**Supplementary Table 1**: Symptom domains with conceptual overlap between CTCAE and EORTC items

| **CTCAE toxicity** | **EORTC item** |
| --- | --- |
| Diarrhea | “have you had diarrhea” |
| Nausea | “have you felt nauseated” |
| Vomiting | “have you vomited” |
| Pain | “have you had pain”  “did pain interfere with your daily activities” |
| Fatigue | “did you need to rest”  “have you felt weak”  “were you tired” |
| Mucositis | “did you have a dry mouth” |

**Supplementary Table 2**: Alignment between CTCAE grades and EORTC scores. Agreement was defined based on previously published literature by Quinten et al., 2011.^17^

| **CTCAE grade** | **EORTC score** |
| --- | --- |
| 0 | 1 (“not at all”) |
| 1 | 2 (“a little”) |
| 2 | 3 (“quite a bit”) |
| 3 or 4 | 4 (“very much”) |

**Supplementary Table 3:** Discordance percentages between clinician-assigned grades and patient-reported symptom severity (CTCAE vs EORTC), with binomial test for predominance of clinician underreporting (clinician-assigned grade lower than patient-reported score). Results shown by arm and timepoint.

| CTCAE toxicities/ EORTC items | Clinician grade lower than Patient score (%) | Clinician grade higher than Patient score (%) | Binominal  p-value | Clinician grade lower than Patient score (%) | Clinician grade higher than Patient score (%) | Binominal  p-value | Clinician grade lower than Patient score (%) | Clinician grade higher than Patient score (%) | Binominal  p-value | Clinician grade lower than Patient score (%) | Clinician grade higher than Patient score (%) | Binominal  p-value |
| --- | --- | --- | --- | --- | --- | --- | --- | --- | --- | --- | --- | --- |
|  | **Middle of Treatment** | | | | | | **End of Treatment** | | | | | |
|  | **Arm A** | | | **Arm B** | | | **Arm A** | | | **Arm B** | | |
| Diarrhea | 17.3 | 0.5 | < 0.001 | 17.9 | 0.9 | < 0.001 | 30.3 | 1.2 | < 0.001 | 24.6 | 0.9 | < 0.001 |
| Nausea | 38.1 | 2.2 | < 0.001 | 25 | 4.8 | < 0.001 | 23.2 | 0.7 | < 0.001 | 18.8 | 0.7 | < 0.001 |
| Vomiting | 10.7 | 2.1 | < 0.001 | 6.9 | 4.2 | 0.02 | 4.6 | 0.5 | < 0.001 | 4.6 | 0.3 | < 0.001 |
| Mucositis | 57.7 | 1.2 | < 0.001 | 52.8 | 1.4 | < 0.001 | 70.8 | 1.2 | < 0.001 | 56.2 | 3.1 | < 0.001 |
| Fatigue | 48.5 | 3.7 | < 0.001 | 36 | 3.3 | < 0.001 | 54.4 | 2.7 | < 0.001 | 43.7 | 3.0 | < 0.001 |
| Pain | 26 | 1.7 | < 0.001 | 23.4 | 4.0 | < 0.001 | 38.2 | 4.6 | < 0.001 | 26.5 | 10 | < 0.001 |

**Supplementary Table 4:** Agreement between clinician-reported CTCAE toxicities and patient-reported EORTC symptom scores, with weighted Cohen’s kappa presented by treatment arm and timepoint for Sweden, Austria and Germany.

| Treatment arm and time-point | Toxicity | No. | Cohen’s Kappa | p-value | No. | Cohen’s Kappa | p-value | No. | Cohen’s Kappa | p-value |
| --- | --- | --- | --- | --- | --- | --- | --- | --- | --- | --- |
|  |  | **Sweden** | | | **Austria** | | | **Germany** | | |
| Arm A, middle of treatment | Diarrhea | 320 | 0.49 | <0.001 | 126 | 0.26 | <0.001 | 220 | 0.26 | <0.001 |
|  | Nausea | 320 | 0.36 | <0.001 | 126 | 0.33 | <0.001 | 221 | 0.36 | <0.001 |
|  | Vomiting | 322 | 0.31 | <0.001 | 125 | 0.31 | <0.001 | 224 | 0.27 | <0.001 |
|  | Mucositis | 318 | 0.22 | <0.001 | 125 | 0.12 | <0.001 | 221 | 0.08 | 0.04 |
|  | Fatigue | 319 | 0.29 | <0.001 | 123 | 0.16 | 0.01 | 211 | 0.12 | 0.01 |
|  | Pain | 318 | 0.30 | <0.001 | 126 | 0.17 | <0.001 | 214 | 0.15 | 0.01 |
| Arm B, middle of treatment | Diarrhea | 351 | 0.17 | <0.001 | 123 | -0.03 | 0.5 | 231 | 0.43 | <0.001 |
|  | Nausea | 349 | 0.24 | <0.001 | 124 | 0.27 | <0.001 | 232 | 0.37 | <0.001 |
|  | Vomiting | 351 | 0.12 | <0.001 | 125 | 0.44 | <0.001 | 233 | 0.46 | <0.001 |
|  | Mucositis | 351 | 0.19 | <0.001 | 121 | -0.004 | 0.86 | 232 | 0.09 | 0.02 |
|  | Fatigue | 349 | 0.27 | <0.001 | 122 | 0.05 | 0.33 | 227 | 0.09 | 0.09 |
|  | Pain | 346 | 0.08 | 0.135 | 123 | -0.02 | 0.77 | 228 | 0.14 | 0.02 |
| Arm A, end of treatment | Diarrhea | 278 | 0.30 | <0.001 | 110 | 0.13 | 0.01 | 199 | 0.21 | <0.001 |
|  | Nausea | 278 | 0.37 | <0.001 | 111 | 0.17 | 0.01 | 201 | 0.33 | <0.001 |
|  | Vomiting | 277 | 0.14 | 0.01 | 111 | 0.2 | 0.02 | 201 | 0.68 | <0.001 |
|  | Mucositis | 278 | 0.08 | 0.06 | 108 | 0.07 | 0.01 | 200 | 0.08 | 0.007 |
|  | Fatigue | 278 | 0.33 | <0.001 | 108 | 0.09 | 0.05 | 197 | 0.16 | 0.001 |
|  | Pain | 273 | 0.38 | <0.001 | 111 | 0.11 | 0.05 | 197 | 0.24 | <0.001 |
| Arm B, end of treatment | Diarrhea | 341 | 0.33 | <0.001 | 118 | 0.07 | 0.24 | 237 | 0.17 | <0.001 |
|  | Nausea | 342 | 0.36 | <0.001 | 119 | 0.20 | <0.001 | 237 | 0.29 | <0.001 |
|  | Vomiting | 342 | 0.24 | <0.001 | 119 | 0.36 | <0.001 | 238 | 0.43 | <0.001 |
|  | Mucositis | 339 | 0.16 | 0.001 | 117 | 0.01 | 0.64 | 232 | 0.02 | 0.57 |
|  | Fatigue | 342 | 0.33 | <0.001 | 117 | 0.07 | 0.2 | 230 | 0.11 | 0.03 |
|  | Pain | 336 | 0.26 | <0.001 | 117 | 0.06 | 0.41 | 234 | 0.22 | <0.001 |
